# Supplementary material for: Influence of the Fatty Acid Metabolism on the Mode of Action of a Cisplatin(IV) Complex with Phenylbutyrate as Axial Ligands
Source: Pharmaceutics. 2023 Feb 16;15(2):677. doi: 10.3390/pharmaceutics15020677 (PMC9967619; doi:10.3390/pharmaceutics15020677)
Supplement: Supplementary file 1 [file pharmaceutics-15-00677-s001.zip › pharmaceutics-2203505-supplementary.pdf]

Supporting information

Table of Content

Figure S1. Cell growth of PhB treated cells. 2
Figure S2. Cisplatin treatment increases nuclei size. 3
Figure S3. Flow cytometry data from CT26 and B16 cells using propidium iodide staining after 24 h treatment. 4
Figure S4. Seahorse experiments of CT26 and B16 cells..5
Table S1. Cell lines used in this manuscript...6

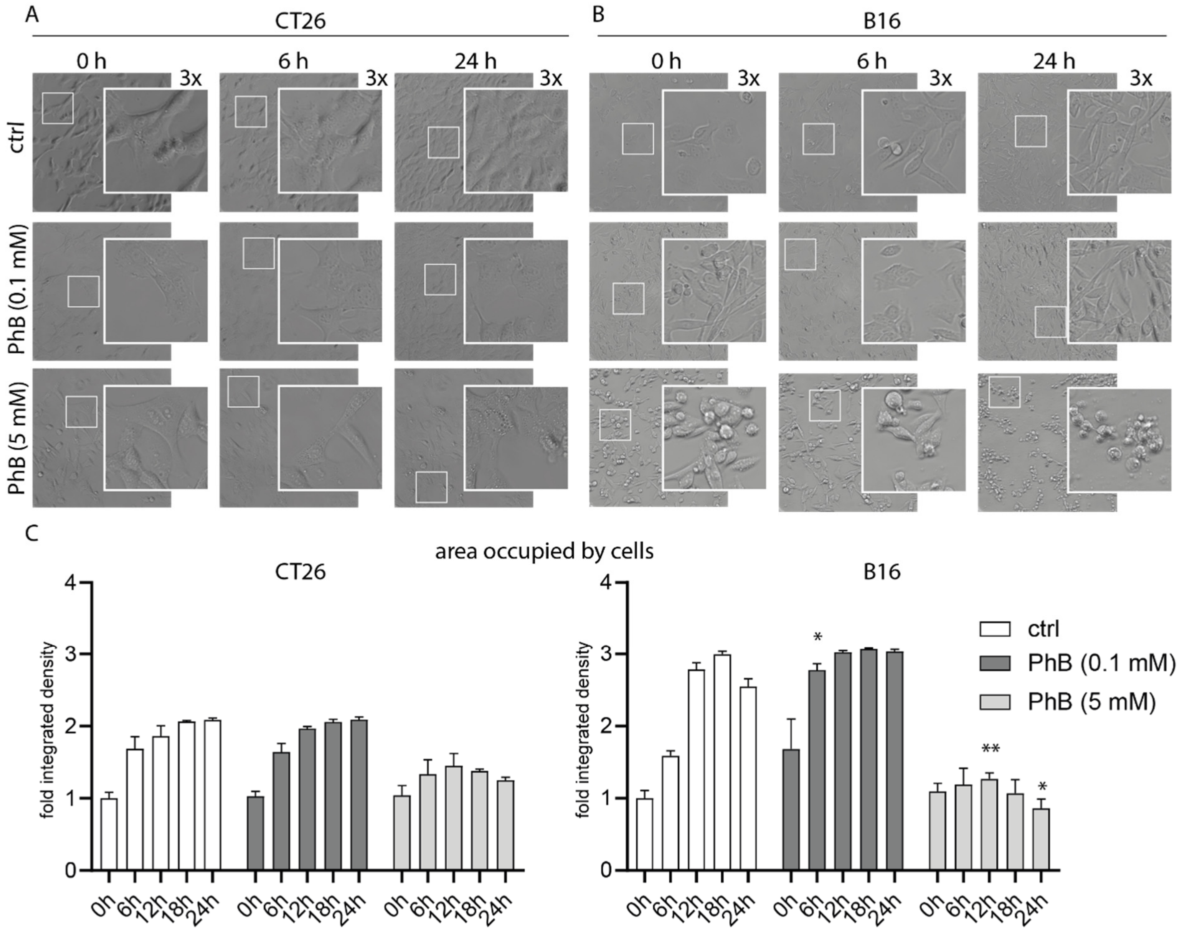

Figure S1. Cell growth of PhB-treated cells. Live-cell images show CT26 (A) and B16 (B) cells treated with PhB after various time points (20x and additional 1.5x magnification). (C) Graph shows quantification of the area occupied by cells from (A) and (B). Bars depict mean ± SD from two representative images (683x683 µm), statistical significance compared to control values was calculated by two-way ANOVA with Sidak's multiple comparison test *<0.05 ** p <0.01.

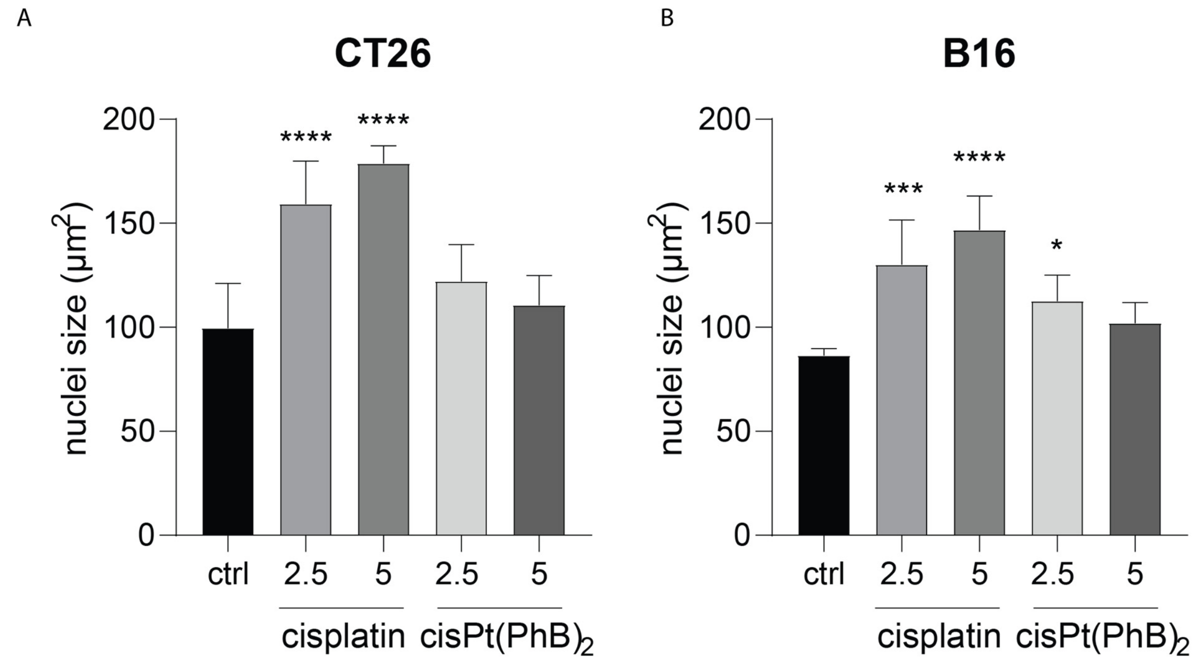

Figure S2. Cisplatin treatment increases nuclei size. Graphs show (A) CT26 or (B) B16 average nuclei size from Figure 4 calculated from five images using imageJ particle analysis. Significance was calculated compared to control by ordinary one-way ANOVA and Dunnett's multiple comparisons test. * $p < 0.05$, *** $p < 0.001$, **** $p < 0.0001$

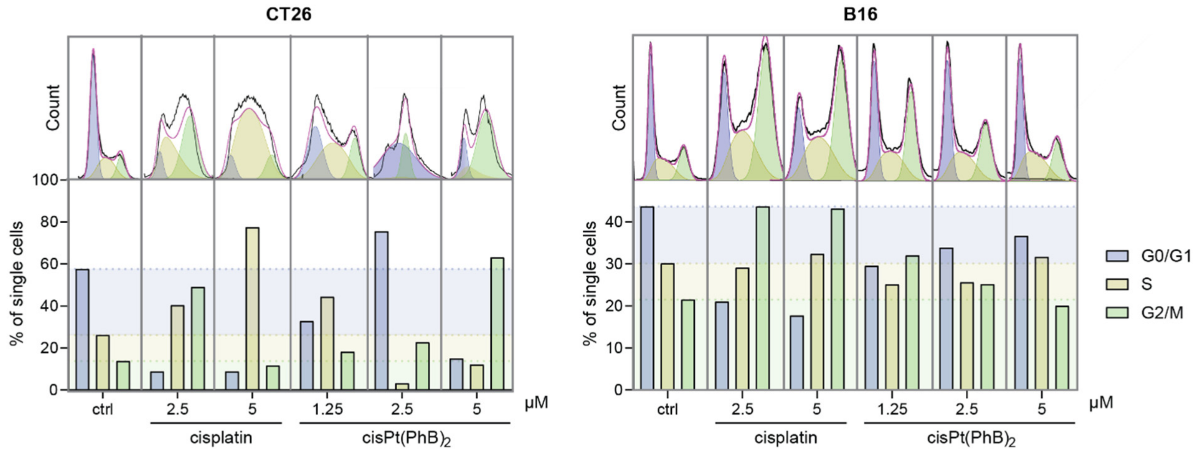

Figure S3. Flow cytometry data from CT26 and B16 cells using propidium iodide staining after 24 h treatment. Upper panel shows histograms from one representative experiment, lower graphs show data as mean from two independent experiments.

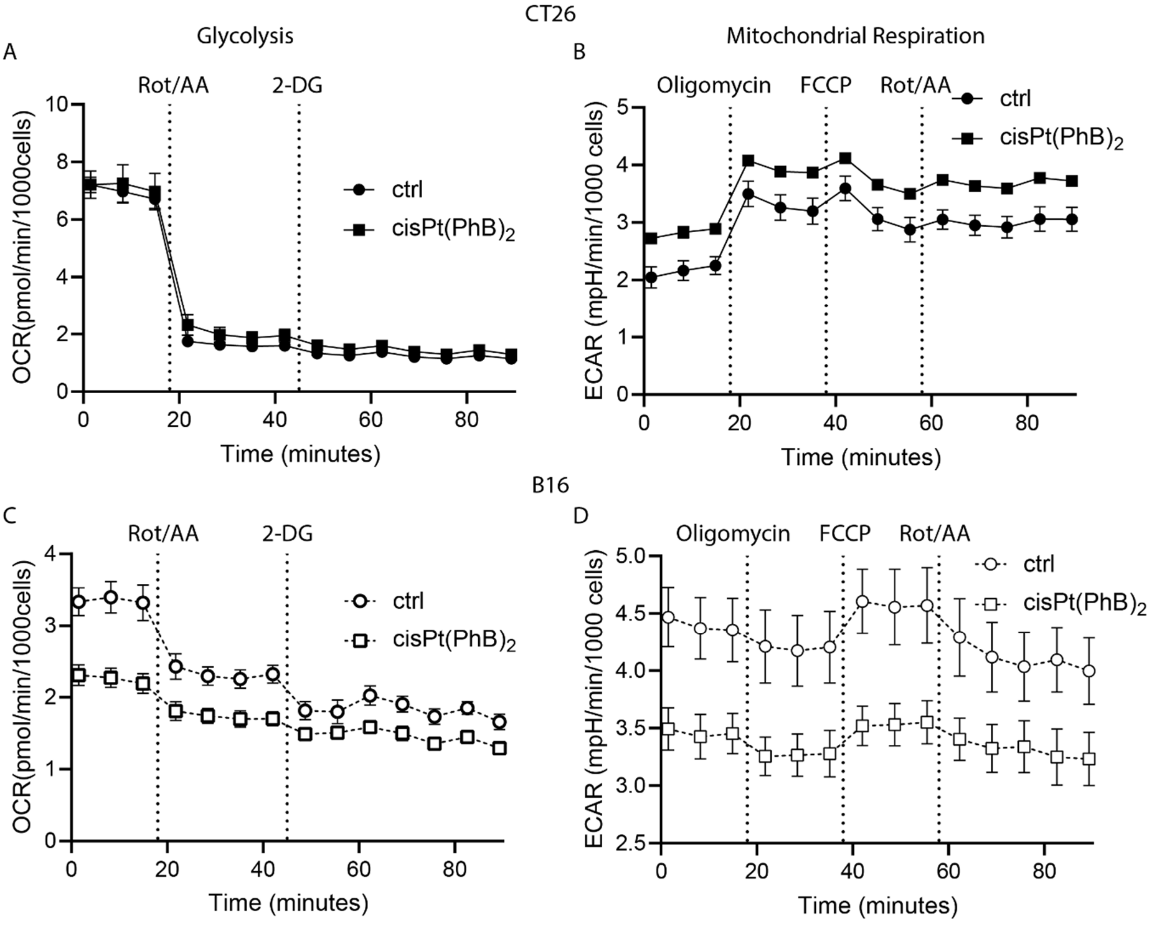

Figure S4. Seahorse experiments of CT26 and B16 cells. Graphs show (A, C) OCR in a glycolytic rate assay and (B, D) ECAR in a mitochondria stress test of CT26 and B16 cells measured in real-time Seahorse experiment under basal conditions and in response to indicated mitochondrial inhibitors. Cells are either untreated or pretreated for 4 h with 5 μM of cisPt(PhB)$_2$.

Table S1. Cell lines used in this manuscript.

Cell line	Species	Origin	P53 status	Source	Medium
HCT116	h	colon carcinoma	Wt	B. Vogelstein (Johns Hopkins University, Baltimore)	McCoy's 5A
HCT116p53KO	h	colon carcinoma	Ko	B. Vogelstein (Johns Hopkins University, Baltimore)	McCoy's 5A
RKO	h	colon carcinoma	Wt	E. Selzer (Medical University Vienna)	McCoy's 5A
RKOp53KO	h	colon carcinoma	Ko	E. Selzer (Medical University Vienna)	McCoy's 5A
SW480	h	colon carcinoma	Mut	ATCC	MEM
CT26	m	colon carcinoma	Wt	ATCC	DMEM/F12 (1:1)
MC38	m	colon adenocarcinoma	Mut	Kerafast, Massachusetts, US	DMEM, 10 µl/ml NEAA, 10 µl/ml pyruvate, 1 ml/100 ml glutamine
A2780	h	ovarian carcinoma	Wt	Merck, Darmstadt, Germany	RPMI-1640
A2780/cisR	h	ovarian carcinoma	Wt	Merck, Darmstadt, Germany	RPMI-1640, 1 µM cisplatin
SKOV3	h	ovarian carcinoma	Mut	ATCC	McCoy's 5A
Capan-1	h	pancreatic carcinoma	Mut	ATCC	RPMI-1640
PANC-1	h	pancreatic carcinoma	Mut	ATCC	DMEM
VM1	h	melanoma metastasis	Mut	Vienna, established at ICR	RPMI-1640
B16	m	melanoma	Mut	ATCC	RPMI-1640
P31	h	mesothelioma	Mut	K. Grankvist (Umeå University, Sweden)	MEM
P31/cisR	h	mesothelioma	Mut	Vienna, established at ICR	MEM, 4 µM cisplatin
MDA-MB-231	h	Breast carcinoma	Mut	ATCC	RPMI-1640

Abbreviations: American Type Culture Collection (ATCC), Institute of Cancer Research (ICR), minimum essential medium (MEM), Dulbecco's modified Eagle's medium (DMEM), DMEM with Ham's F-12 basal media (DMEM/F12), non-essential amino acid (NEAA), Roswell Park Memorial Institute (RPMI)
